# Mental Health First Aid training for Chile and Argentina: protocol for a cluster randomised controlled trial

**DOI:** 10.1136/bmjopen-2025-105308

**Published:** 2026-02-12

**Authors:** Martin Agrest, Esteban Encina-Zúñiga, Sara Ardila-Gómez, Marina Ayelén Fernandez, Raquel Gabriel, Jessica Bargamian, Rubén Alvarado, Amy J Morgan, Claire M Kelly, Nicola Reavley

**Affiliations:** 1 Proyecto Suma, Buenos Aires, Argentina; 2 Instituto de Investigaciones, Universidad de Buenos Aires Facultad de Psicología, Buenos Aires, Argentina; 3 Faculty of Social Sciences, University of Chile, Santiago, Chile; 4 School of Public Health, Faculty of Medicine, University of Chile, Santiago, Chile; 5 Consejo Nacional de Investigaciones Cientificas y Tecnicas, Buenos Aires, Argentina; 6 Instituto de Investigaciones en Psicología, Universidad de Buenos Aires Facultad de Psicologia, Buenos Aires, Argentina; 7 Dartmouth College, Hanover, New Hampshire, USA; 8 Department of Public Health, Universidad de Valparaiso, Valparaíso, Chile; 9 Melbourne School of Population and Global Health, The University of Melbourne, Melbourne, Victoria, Australia; 10 Mental Health First Aid Australia, Parkville, Victoria, Australia

**Keywords:** Health Literacy, Social Support, MENTAL HEALTH

## Abstract

**Introduction:**

Community support for individuals with mental health problems is a global public health issue. Poor mental health literacy and high levels of stigmatising attitudes among the general population can hinder both help-seeking behaviours and limit the capacity of community members to provide support to people experiencing mental health challenges. The Mental Health First Aid (MHFA) training course was created to educate community members to provide initial help towards a person developing a mental health problem. MHFA training has spread to high-income countries, but there is relatively little research on cultural adaptation to lower-resource settings. This study aims to fill that gap and is the first cluster randomised controlled trial (cRCT) assessing the effectiveness of MHFA training in Chile and Argentina.

**Methods and analysis:**

The study involves a two-arm wait-list cRCT with 240 participants (120 in each country). The study will be conducted in three settings in each of Chile and Argentina (eg, universities, health services and workplaces). Two clusters per setting in each country will be paired and randomly allocated to the intervention (the MHFA training for Chile and Argentina) or the wait-list control group. Participants in the intervention arm will be asked to complete questionnaires at baseline (T1), after training completion (T2) and 6 months after completion (T3), with control arm participants completing data collection at corresponding time points. The primary outcome will be intended support towards someone experiencing a mental health problem or experiencing a mental health crisis. Secondary outcome measures will include the ability to recognise depression and psychosis in vignettes; beliefs about the helpfulness of different types of interventions and helping actions, confidence in providing MHFA and stigmatising attitudes towards a person with depression or psychosis. Findings will demonstrate whether the culturally adapted MHFA training for Chile and Argentina can effectively enhance intended support, knowledge, attitudes and supportive actions towards other individuals within the community.

**Ethics and dissemination:**

Ethics approval has been granted by the Human Research Ethics Committee at the University of Melbourne (Australia), Proyecto Suma (Argentina) and the University of Chile (Chile). Dissemination will be via academic publications and conference presentations. These will also be made available to participants and other interested parties on request.

**Trial registration number:**

ISRCTN63724445.

**STRENGTHS AND LIMITATIONS OF THIS STUDY**

- This represents the first study assessing the effectiveness of a Mental Health First Aid training course in Latin America, equipping community members with the skills to recognise and support individuals experiencing mental health problems or crises.
- Findings from Chile and Argentina will provide valuable insights to inform the dissemination of Mental Health First Aid training in these and other comparable countries.
- Cross-country comparisons should be interpreted with caution due to contextual and health system differences between countries.
- Individuals with limited literacy will not be included, as the study relies on self-completed questionnaires, which may limit generalisability.
- Further research will be needed to evaluate the impact of Mental Health First Aid training on recipients of assistance provided by trained individuals.

## Introduction

 Despite notable differences between Argentina and Chile regarding their mental health reforms,^1^ these two Latin American countries have made considerable progress towards addressing mental health problems through community-based approaches and a shift away from institution-based care.^2^,^4^ Although at different rates, both countries have prioritised mental healthcare through the implementation of specific mental health legislation^5 6^ and mental health plans.^7 8^ Adding to this, they have established programmes aiming to enhance suicide prevention^9^ and to tackle the psychological consequences of potentially traumatic events.^10^,^12^ However, these strategies, as in other countries, have not been properly evaluated and face multiple hurdles that coexist with high levels of stigma toward individuals with mental health problems, persistent treatment gaps and the limited sensitivity of healthcare services to individuals’ needs and preferences. This situation calls for additional progress and fresh initiatives.

In Argentina, the latest national mental health survey estimated that the lifetime prevalence of any mental disorder among adults was 29.1%,^13^ while in Chile it has been estimated at 31.5%.^14^ Moreover, it has been estimated that following the COVID-19 pandemic, the prevalence of common mental health issues in the region may have risen.^15^ It is increasingly likely that individuals will have first-hand experience with mental health problems, whether personally, through a family member or via a close acquaintance.

In Argentina and Chile, and Latin cultures more broadly, informal networks have played a crucial role in the provision of help, with an emphasis on family and friends being involved in supporting those in need.^16^,^20^ Furthermore, the integration of these informal networks with formal networks of care, such as the healthcare system, has garnered significant attention over the years.^21 22^ Nonetheless, both formal and informal networks, as well as their interconnections, have significant potential for further development.

The availability of these programmes and services does not preclude Argentina and Chile from potentially benefitting from interventions that align with their cultural emphasis on support from family and friends, particularly those that enhance mental health literacy (ie, understanding of mental health issues and treatment options) and specifically address common challenges such as problem alcohol use, depression and anxiety disorders. As community members are highly likely to have contact with a person with a mental health problem, programmes that educate them to provide early help towards a person developing a mental health problem, experiencing a worsening of an existing mental health problem or in a mental health crisis, may contribute to improving access to services, mental health outcomes and also enhance social support.^23^

The Mental Health First Aid (MHFA) programme has emerged as one of the most widely adopted and evidence-based interventions designed to enhance community responses to mental health concerns, particularly in high-income countries.^24^ It equips members of the public with the skills to offer initial assistance to someone showing signs of a mental health issue or experiencing a mental health crisis. This support is intended to bridge the gap until appropriate professional care becomes available or the situation stabilises.^25^ Delivered via structured training programmes, MHFA was initially developed by the Australian non-profit organisation MHFA International. Since its inception, the programme has expanded significantly, with affiliated courses now offered in nearly 30 countries and several million individuals trained globally. Evaluations of MHFA training indicate positive outcomes, including enhanced mental health literacy, greater confidence in offering support, reduced stigma and improved helping behaviours among participants.^26^ Despite its global reach, MHFA has not yet been rolled out in Latin America, and questions remain regarding the cultural relevance of the training in this context. Some initial work has been undertaken to adapt MHFA for Latin communities in the USA,^27^ but further exploration is needed to assess its suitability across diverse cultural and healthcare environments in the region.

### The current study

This study represents the first evaluation of culturally adapted MHFA training tailored for Latin American contexts, specifically Chile and Argentina. It is a collaborative effort involving the University of Melbourne, MHFA International, Proyecto Suma (Argentina) and the University of Chile. The paper outlines the protocol for a cluster randomised controlled trial (cRCT) designed to assess the effectiveness of the adapted MHFA programme across various settings where participants are likely to encounter individuals experiencing mental health challenges. By including a range of real-world contexts, the study aims to enhance the generalisability of the findings across diverse populations and service environments in these countries. The reporting of the protocol follows Standard Protocol Items: Recommendations for Interventional Trials (SPIRIT) guidelines, with the SPIRIT checklist provided as online supplemental material.^28^

## Methods and analysis

### Study design

A two-arm cRCT will be used to evaluate the effectiveness of MHFA training in Chile and Argentina. A cluster will be defined as a single training group (approximately 20 participants) recruited within one organisation in a given setting and country. Within each setting and country, two clusters will be paired, with one allocated to the intervention and one to the wait-list control group. The settings include universities (undergraduate alumni), healthcare personnel and corporate workplaces. Participants will be compared with a control group that will receive the training course after the study has been completed. To facilitate engagement and retention, the local research team will approach organisations that have expressed interest in receiving MHFA training. The settings were selected because they represent contexts in which community members are likely to encounter individuals experiencing mental health problems, and because MHFA is commonly implemented in similar settings internationally, supporting future scalability and implementation.

The trial aims to assess the impact of MHFA training for Chile and Argentina on: intended support for someone developing a mental health problem or in a mental health crisis; the capacity to recognise in a vignette whether someone is experiencing depression or psychosis; beliefs about the helpfulness of interventions for a mental health problem (ie, depression or psychosis); helping actions taken to assist a person in these situations; confidence in providing MHFA; stigmatising attitudes; and desire for social distance from a person with an affective mental health problem (ie, depression) or psychosis.

**Figure 1:**
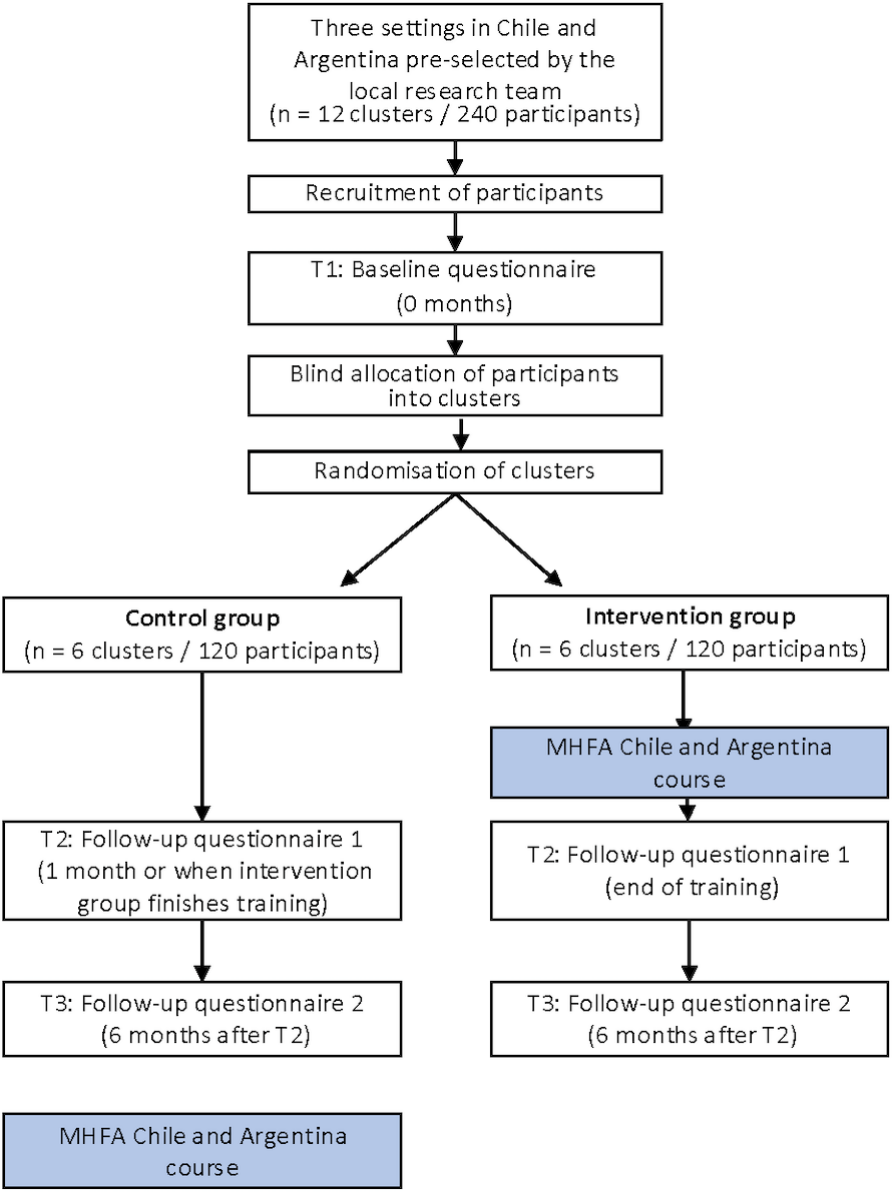
Trial schema showing cluster randomisation and assessment intervals for both arms. MHFA, Mental Health First Aid.

**Figure 2:**
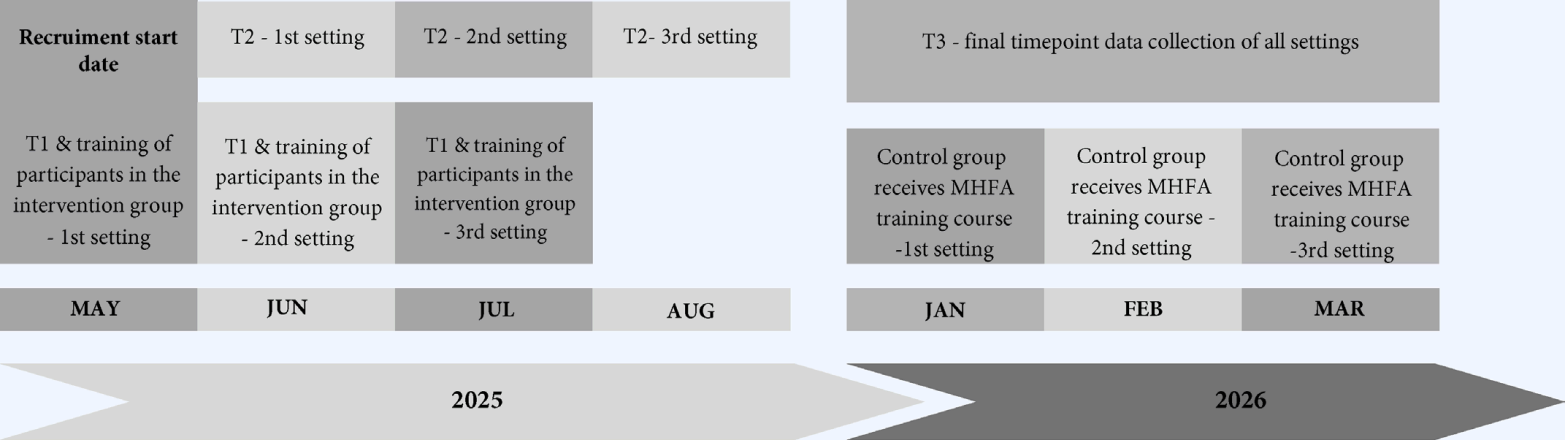
Trial schema showing the timeline with enrolment and assessment points. MHFA, Mental Health First Aid.

The trial coordinator will recruit participants in each country. Clusters from each setting will be paired and randomly allocated to the intervention (MHFA training in Chile and Argentina) or the wait-list control group, with a 1:1 allocation. Figure 1 illustrates the trial design, and figure 2 shows the timeline with enrolment and assessment points. The course will be delivered face-to-face.

The trial is registered with the International Standard Randomised Controlled Trial Number Registry (ISRCTN63724445) and will be conducted and reported in accordance with the Consolidated Standards of Reporting Trials guidelines, with extension for cluster trials.^29^,^31^ The outcomes pertain to the individual participant level.

### The intervention

MHFA training for Chile and Argentina is a major adaptation based on the Australian Standard MHFA 12-hour course as well as the Chinese experience of adapting this course, for which several guidelines have been ‘blended’ into a more general approach to different mental health problems and crises.^25^ The Australian training programme is based on a five-step action plan: Assess, Listen, Give support and information, Encourage appropriate professional help, Encourage other supports.^25^ However, the Chinese adaptation included a slightly modified four-step action plan more suitable for countries where mental health literacy has more room for improvement and pathways to mental healthcare are more complicated. The new action plan was renamed as Recognise, Engage, Keep safe and Support.^32^

The adaptation of the MHFA course materials for use in Chile and Argentina was carried out by MA, EE-Z, RG and CK. Similar to the Australian and Chinese versions, the Latin American MHFA courses comprise a comprehensive package including a regionalised manual, instructor guidelines, presentation slides, videos and interactive activities designed to support skill development. The curriculum content was shaped by five Delphi expert consensus studies conducted between 2020 and 2023 in both countries. These studies involved input from mental health professionals, individuals with lived experience and carers, and provided evidence-based, culturally relevant guidance on appropriate first aid responses—particularly in relation to supporting someone showing signs of developing harmful alcohol use,^33^ depression,^34^ at risk of suicide,^35^ psychosis^36^ or at risk after a potentially traumatic event.^37^ The research team further adapted the Chinese MHFA Action Plan to ensure its suitability for Argentina and Chile. This process drew on suggestions from local experts given during the Delphi studies. In addition, actions not endorsed in Argentina and Chile during the adaptation process were excluded from the manual. The manual was organised in three chapters outlining information on mental health and mental health problems or crises, the basic principles of MHFA and a final chapter outlining each of the four actions. Based on this manual, the PowerPoint slides were changed accordingly after being translated from English into Spanish. Some of the original artwork featured in the slides was replaced with pieces created by people with lived experience from Proyecto Suma (Argentina). The instructor’s teaching notes were redeveloped based on the PowerPoint slides. Spanish subtitles were also created for seven of the Australian MHFA videos to ensure accessibility for participants who do not speak English. The training course comprised 4 weekly sessions each lasting 3 hours (12 hours in total).

The MHFA training course was pilot-tested in Argentina and Chile to inform final refinements to the intervention. Feedback collected from participants during pilot sessions was used to optimise course content, structure and timing, ensuring that all topics could be adequately covered within the planned course duration. Based on this feedback, minor modifications were made to reduce redundancy, consolidate case material, clarify the organisation of content and tailor information on available professional support to local contexts.

Trainers from both countries, all with a background in clinical psychology, jointly received initial training and supervision from CMK and NR during the materials revision process and committed to delivering all courses in pairs in their respective countries throughout the study. In addition, a fidelity assessment tool was developed by the local teams, in consultation with NR and the MHFA International representative (CMK), to ensure comparability across courses and between sites.

### Randomisation

Clusters will be matched by setting (university, healthcare or workplace) and then randomly allocated to either the intervention or control condition. Local trial managers in Argentina and Chile will be responsible for recruiting participants into their respective clusters, using convenience sampling and taking into account degree programmes and areas of employment to achieve a reasonable balance in years of education, so that participants in the intervention and the wait-list groups are as comparable as possible. Random allocation of clusters will be conducted by a statistician, who will be blinded to any identifying details and will use a random sequence generator within the Stata software package to assign groups. Due to the nature of the intervention, blinding of participants and research staff post-randomisation will not be possible. However, allocation will remain concealed from all parties until after participants have enrolled in the study.

### Setting and participants

#### Recruitment of clusters

The local research teams in Chile and Argentina will contact alumni offices at universities, personnel departments at healthcare centres and human resources managers in large companies to invite individuals to participate in the study. Representatives from these organisations will be interviewed and provided with a Plain Language Statement prior to participant recruitment and randomisation. Multiple meetings with representatives from each setting are planned in order to minimise the influence of organisational interests on the recruitment of participants. We anticipate conducting one training course per setting in each country with the intervention group (and another at the end with the wait-list group). Each cluster is expected to include approximately 20 participants. No formal pairing criteria will be applied; however, the relative homogeneity of each setting and consideration of education level are intended to contribute to comparability between groups. In addition, potential contamination of groups will be considered (eg, participants in the intervention group sharing what they learnt or training materials with participants in the control group). For example, students pursuing the same degree will be allocated to the same cluster, and participants from the same (or different) areas or facilities will likewise be grouped accordingly, in order to minimise the possibility of participants from different groups having regular contact in the course of their study or work. A summary of the number of clusters per arm, assumed cluster size, cluster types and pairing approach is provided in online supplemental table 1.

#### Recruitment of participants

All participants (adults aged 18 and over) in each setting in both countries will be eligible. Only individuals with prior formal training in mental health (eg, mental health professionals, advanced students of psychology or medicine or training in psychological first aid) will be excluded. Due to the written nature of the course materials and study instruments, participants will be limited to individuals for whom the materials are accessible in their current format (eg, those with literacy levels at or above middle school). Potential participants will receive information about the study through key contacts at each setting. Individuals can express their interest in participating either through an online registration and consent platform or by reaching out directly to a local representative or the research team via WhatsApp or email. The local trial team will provide all prospective participants with a Plain Language Statement outlining the study, and informed consent will be obtained from those who agree to take part in the trial.

### Data collection and measures

All participants will be asked to complete a baseline questionnaire before the MHFA training course in each site begins (T1), follow-up questionnaires when the training course is finished (T2) and a final questionnaire 6 months after the training ends (T3). Participants in the control group will also complete these questionnaires at corresponding time intervals and will be able to enrol in the course after completing the T3 questionnaire. Participants will receive up to three questionnaire reminders either by email or WhatsApp. Participants in the wait-list arm may choose not to take the course, and this will not affect their participation in the study.

Participants in the intervention group will be advised not to share training materials (eg, the training manual) with others for at least 6 months as they may be unaware whether those individuals are allocated to the control group. Participants in the wait-list arm will be explicitly asked about any prior exposure to the training curriculum before receiving the training; the proportion of participants reporting such exposure and its distribution across clusters will be considered in the interpretation of the findings.

Data will be collected through an online questionnaire platform, Qualtrics. Participants will receive a personal email link. We will attempt to maximise cluster and participants’ retention through collaboration with local representatives and reminders to complete questionnaires.

Socio-demographic information (age, gender, employment status, occupational category, marital status and level of education and whether the respondent manages staff) will be sought at T1 only. Later questionnaires will be identified and linked to T1 responses through a unique identifier that will be generated for each participant.

Anticipated difficulties during the trial include the recruitment of participants and their retention during the follow-up period. We expect to overcome this by offering reimbursement at T2 and T3 for completion of all questionnaires. Additionally, we aim to counterbalance potential attrition by providing participants with certificates that validate their training on completion of the trial. These certificates may serve as a valuable credential to formalise their skills and knowledge. Furthermore, we have allowed for 30% attrition in our power calculation.

Performance bias will be minimised by ensuring that baseline (T1) questionnaires are completed before participants are informed of their allocation to the intervention or the control group. In addition, invitations and reminders will be identical across groups at all time points. At T2 and T3, participants will be reminded to carefully complete the questionnaires. Research staff involved in participant contact will use standardised and neutral messaging with no differentiation by study arm. To reduce the influence of inattentive responding, response times will be monitored. Questionnaires completed in less than 5 min (approximately a quarter of the anticipated average completion time of 20 min) will be flagged. The proportion of such responses and their distribution across study arms and time points will be reported, and their potential influence on the results will be explored.

Outcomes are assessed using self-report measures, and self-report bias (eg, social desirability) cannot be ruled out. However, the instruments have been previously piloted and used in similar populations, demonstrating acceptable reliability.^38^ Repeated measurements over time will also allow the examination of consistency in response patterns, which will be reported.

#### Primary outcome

##### Intentions to provide Mental Health First Aid (Mental Health Support Scale— Intended)

Intentions to provide MHFA will be assessed with the Mental Health Support Scale (MHSS) that has been validated for Argentina and Chile.^38^ An English-language version of this scale was developed by drawing on actions from the MHFA guidelines for English-speaking countries.^39^ This was then culturally adapted for Argentina and Chile. Respondents are asked to imagine a person they know well who is experiencing a mental health problem, experiencing a worsening of an existing mental health problem, or is in a mental health crisis (eg, they are suicidal). Subsequent questions cover the likelihood of undertaking different actions to help the person, on a 5-point scale (very unlikely to very likely). The scale consists of 21 items with two subscales: recommended actions (16 items) and non-recommended actions (5 items). Higher scores indicate better mental health support. Internal consistency was excellent (omega=0.93 and 0.88), the scales discriminated between those with and without MHFA training.

### Secondary outcomes

#### Recognition of depression and psychosis

Given the high prevalence of depression and the likelihood of encountering individuals with these symptoms in the trial settings,^13 14^ a vignette featuring a person named ‘J’ who meets the Diagnostic and Statistical Manual of Mental Disorders, 5th Edition (DSM-5) criteria for major depression will be used. Participants will be asked the following question—‘What, if anything, do you think is wrong with J?’—to assess their ability to recognise whether J may be experiencing depression (with open-ended responses). Any response with ‘depression’ or ‘depressed’ will be coded as correct. The vignette is based on one used in previous English-language MHFA trials. It has demonstrated 2 month test–retest reliability of 0.64 (0.49–0.76).^26^

To ensure accuracy of translation, the vignette was translated into Spanish independently by two bilingual translators (native Spanish speakers); the translations were compared item by item and, after reaching consensus, were back-translated into English by a bilingual native English speaker. The back-translation was compared with the original version and no substantive differences were identified. Minor discrepancies were discussed as a group and the final Spanish version was agreed by consensus among all bilingual translators involved in this process.

Given the high level of disability associated with psychosis and the critical importance of its early detection, a vignette describing a person (‘A’) with psychotic symptoms consistent with DSM-5 criteria for schizophrenia, which underwent an identical translation process, will also be used, with responses coded as ‘correct’ if they include mention of ‘psychosis’ or ‘schizophrenia’. Cognitive interviewing was not conducted as a separate step; however, the vignettes were reviewed by the research team and administered during pilot testing of the course, during which no major difficulties in comprehension were identified. Both vignettes will use a gender-neutral format.

#### Beliefs about treatment and help for a person with depression or psychosis

These will be assessed using an adapted version of a 16-item scale used in an MHFA trial in Australia,^40^ which is based on a consensus between Australian clinical psychologists, psychiatrists and general practitioners (GPs) established by a national survey.^41^ After each vignette, respondents will be presented with sources of potential help for depression or for psychosis and four response options for each (‘helpful’, ‘unhelpful’, ‘neither’ and ‘don’t know’). For depression, correct responses will be considered when participants select the ‘helpful’ option in each of the following cases: a psychiatrist; a psychologist; a typical family GP or doctor; psychotherapy; antidepressants; reading about people with similar problems and how they have dealt with them; becoming more physically active; and cutting out alcohol altogether. They will also score 1 point for rating ‘dealing with the problem alone’ as ‘harmful’.^42^ For psychosis, correct responses include a typical family GP or doctor; a psychiatrist; taking antipsychotic medications and cutting out alcohol as ‘helpful’, and rating ‘dealing with the problem alone’ as ‘harmful’.

#### Confidence in providing Mental Health First Aid for a person with depression or psychosis

Participants will also be asked to rate their confidence in supporting the person in each of the vignettes (1=not at all confident to 7=extremely confident).

#### Helping actions taken to assist a person with a mental health problem

Participants will be asked about any helping actions taken to assist a person who is at risk of a mental health problem or crisis, using the MHSS-provided version that has been adapted and validated for Chile and Argentina. This scale has been shown to be a reliable and valid measure of help provided to a person the respondent knows well.^39^ It consists of 10 items (8 recommended, 2 not recommended) with a response scale of ‘Yes’ or ‘No’. Higher scores indicate better mental health support. Each factor of the MHSS provided for Argentina and Chile showed excellent internal consistency and the Recommended factor discriminated between those with and without mental health training.^38^

#### Desire for social distance from a person with depression and from another person with psychosis

Participants desire to avoid contact with a person with depression or with psychosis will be measured for each vignette. The Social Distance Scale includes five items measured on a 4-point Likert scale (1 =‘yes, definitely’ to 4=‘no, definitely not’).^43^ The scale asks: ‘Would you be happy to: 1) move next door to J/A? 2) spend an evening socializing with J/A? 3) make friends with J/A? 4) work closely with J/A on a project at work? 5) have J/A marry into your family?’ The measure has shown excellent reliability with α=0.88 in community questionnaires in Australia,^44^ and an acceptable reliability (α=0.78) in Chile.^45^ Furthermore, it has been extensively used in Argentina and Chile.^46^,^48^

#### Personal stigmatising attitudes about an individual with depression and another individual with psychosis

For each of the vignettes, participants will be asked to what extent they agree with nine statements designed to measure stigmatising attitudes (1=strongly disagree and 5=strongly agree). The stigma items will be based on the Depression Stigma Scale^49^ and a Chilean version of the scale used with adolescents.^50^

The scale includes the following statements: (1) People with a problem like [initials] could snap out of it if they wanted, (2) A problem like [initials]’s is a sign of personal weakness. (3) A problem like that of [initials] is not a real medical illness, (4) People with a problem like [initials]’s are dangerous to others, (5) It is best to avoid people with a problem like [initials] so that you do not develop this problem, (6) People with a problem like [initials] are unpredictable, (7) If I had a problem like [initials]’s I would not tell anyone, (8) I would not employ someone if I knew they had a problem like [initials]’s in the past and (9) I would not like my children to be looked after by a person like [initials]. The ‘initials’ will refer to a person with psychosis in the first place, and then would refer to a person with depression. The items form two scales ‘Weak not sick’ and ‘Dangerous/Unpredictable’ which have previously been shown to demonstrate high internal consistency in the English-language version.^44^

#### Course quality and satisfaction

In the first follow-up questionnaire (T2), participants in the intervention group will be asked about their satisfaction with the course and their opinions on its quality.^51^ A translated and adapted version of this questionnaire was used in the pilot MHFA training in Argentina and Chile. It includes sixteen items rated on a 4-point or 5-point Likert scale, assessing the usefulness, novelty and relevance of the content, as well as the course duration and participants’ preferences regarding different components (eg, manual, PowerPoint slides, videos and activities). Additionally, two open-ended questions explore which aspects of the course were most helpful and whether any improvements could be made.

The selection of intentions to provide MHFA as the primary outcome is consistent with prior evaluations of MHFA training.^32 52^ In line with MHFA being conceptualised as an intervention targeting mental health literacy, which comprises multiple distinct components,^26 53^ several secondary outcomes are included to capture these dimensions. These secondary outcomes will be treated as exploratory, and findings will be interpreted cautiously in light of the increased risk of type I error associated with multiple comparisons.

#### Power and sample size

According to the Shiny cluster randomised trial (CRT) calculator,^54^ with six clusters per arm (12 in total) the study will have 90% power, at a two-sided 5% significance level, to detect a standardised mean difference of 0.5 in intentions scores between intervention and control groups at 6-month follow-up. In the absence of pilot data, the sample size calculation assumed a conservative intracluster correlation coefficient of 0.05, equal cluster sizes, a correlation of 0.5 between individual measurements over time and a 30% loss to follow-up. The assumed effect size was informed by a meta-analysis of MHFA training, which found pooled effect sizes for intentions were d=0.75 immediately post training and d=0.55 up to 6 months after training.^26^

#### Data monitoring and management

Trial coordinators will be based at Proyecto Suma (in Argentina) and at the University of Chile (in Chile). They will be responsible for the day-to-day running of the project and data management. Global oversight will be handled by NR. Data will be held in a secure, password protected database hosted by the University of Melbourne servers. Only the research team will have access to the data. A data monitoring committee will not be formally established, and interim analyses are not planned, as the study is not expected to involve any serious adverse outcomes. Prior research involving similar mental health interventions has reported low levels of participant distress and high levels of satisfaction, indicating minimal risk associated with participation in this type of research.^55 56^ Notwithstanding, participants will be purposefully asked about any distress experience during the survey completion, will be referred to available local resources and have the option to be contacted by a mental health professional (from Proyecto Suma, in Argentina and from the University of Chile, in Chile).

Participants will be required to register for the trial through an online platform, where they will provide their full name, WhatsApp number and email address. A unique identification code will be assigned to each participant to facilitate data linkage across different time points. At each study site, the research team will maintain a separate and secure record mapping participants’ personal information to their respective codes. Participants will receive a link to an online questionnaire via email (or WhatsApp, if requested), connected to their assigned code. Only de-identified data will be deposited in a data repository (eg, Figshare) to ensure participant confidentiality. NR, AJM, MA and EE-Z will have access to the final data set after completion of the trial. However, NR and AJM will remain blind to arm allocation until completion of the statistical analysis.

#### Statistical analyses

Statistical analyses will be conducted by a researcher not involved in participant recruitment or intervention delivery and blinded to allocation, following intention-to-treat principles; all participants who consent to participation will be included in the analysis, regardless of intervention completion or follow-up responses. Outcomes will be analysed at the individual level (rather than cluster level). Primary and secondary data will be analysed using mixed-effects models for continuous and binary outcome variables, incorporating interactions between group and measurement occasions. This method takes into consideration the data’s hierarchical structure (ie, the correlation of measurement occasions within participants and within clusters) and uses maximum likelihood methods to generate unbiased estimates even when some participants have missing data, provided that the data are missing at random. Country and setting will be added as fixed effects. Predictors of missingness will be examined using logistic regression, and variables linked to missingness will be added to models as fixed effects. Contrasts will compare change between groups from T1 to T2 and T3. Unless there are substantial imbalances at baseline, between-group effect sizes (Cohen’s d) will be calculated by standardising the differences between follow-up scores by the pooled SD. Otherwise, Cohen’s d will be calculated by dividing the mean change in each group by the pooled SD at baseline. Statistical analyses will be conducted using Stata V.19.^57^

### Patient and public involvement statement

Individuals with lived experience of mental ill-health, either personal or as informal caregivers, were involved as members of expert panels advising on the suitability of mental health first aiders receiving (or not receiving) training on specific topics during the development of the guidelines that informed the cultural adaptation of the MHFA training course.

## Ethics and dissemination

Ethics approval has been obtained from the Human Research Ethics Committee at the University of Melbourne (Reference: 2024-30813-59250-3, 17 October 2024, V.3), the Ethics Committee of Proyecto Suma (#2024–14006) and by the Ethics Committee of the University of Chile (#74-89/2024). Any substantial amendment to the protocol will be submitted for ethical review as required.

Dissemination will be via academic publications and conference presentations. These will also be made available to participants and other interested parties on request. The questionnaire used for data collection will likewise be available on request. The university website and MHFA International’s dissemination channels will also be used. Authorship will follow established international guidelines for responsible research conduct.

## Discussion

Findings from this trial will examine whether MHFA training for Chile and Argentina enhances intended support, knowledge, attitudes and supportive actions towards a person developing a mental health problem or experiencing a mental health crisis in these countries. Since there is no prior experience with delivering an MHFA training in these countries, except for the pilot experience mentioned above, the current trial addresses a major gap in knowledge. Furthermore, given their shared historical and cultural roots, coupled with their divergent paths in mental health reform, Argentina and Chile present a compelling opportunity for a collaborative endeavour. By studying the effectiveness of MHFA training across both nations, we hope to gain valuable insights into the applicability and potential adaptations required for such interventions to succeed in diverse societal contexts. Ultimately, this may benefit the well-being of people with mental health problems in Chile and Argentina, strengthen the connection between formal and informal care while enhancing informal support, and reduce the related burden of disease associated with mental health in these (and potentially other) countries.

## Supplementary material

10.1136/bmjopen-2025-105308 online supplemental file 1

10.1136/bmjopen-2025-105308 online supplemental file 2
