# Supplementary material for: Mental Health First Aid training for Chile and Argentina: protocol for a cluster randomised controlled trial
Source: BMJ Open. 2026 Feb 12;16(2):e105308. doi: 10.1136/bmjopen-2025-105308 (PMC12911734; doi:10.1136/bmjopen-2025-105308)
Supplement: online supplemental file 1 [file bmjopen-16-2-s001.pdf]

Supplmentary Table 1. Summary of cluster design characteristics

Number of clusters per arm	6
Assumed cluster size	20 participants
Cluster types	University students, healthcare personnel, corporate workplace
Pairing method	No formal pairing
