# Supplementary material for: Mental Health First Aid training for Chile and Argentina: protocol for a cluster randomised controlled trial
Source: BMJ Open. 2026 Feb 12;16(2):e105308. doi: 10.1136/bmjopen-2025-105308 (PMC12911734; doi:10.1136/bmjopen-2025-105308)
Supplement: Supplementary file 3 [file bmjopen-2025-105308.reviewer_comments.pdf]

PEER REVIEW HISTORY

BMJ Open publishes all reviews undertaken for accepted manuscripts. Reviewers are asked to complete a checklist review form and are provided with free text boxes to elaborate on their assessment. These free text comments are reproduced below.

ARTICLE DETAILS

Title (Provisional)

Mental Health First Aid training for Chile and Argentina: Protocol for a cluster randomised controlled trial

Authors

Agrest, Martin; Encina-Zúñiga, Esteban; Ardila-Gómez, Sara; Fernandez, Marina A. Ayelén; Gabriel, Raquel; Bargamian, Jessica; Alvarado, Rubén; Morgan, Amy J.; Kelly, Claire M.; Reavley, Nicola

---

VERSION 1 - REVIEW

**Reviewer**	**1**
**Name**	**Pham, Lily**
**Affiliation**	**The University of Sydney School of Pharmacy**
**Date**	**26-Jun-2025**
**COI**	**None**

This protocol is generally clear in its adherence to SPIRIT reporting guidelines and its potential impact within the mental health and academic space.

Some feedback on the protocol is as follows:

• Page 2 – Misspelling of strengths in ‘strengths and limitations of this study’.

• Page 2- some reflection on the potential limitations on the study and how they would be mitigated could add to the richness of this protocol

• Introduction page 2, Line 34 – would be good to mention the types of programs that are/were available, what they offer, their measurable impacts (if any) and how they contribute to mental health care.

• Page 3 Line 28 – embed the link to MHFA as a reference, if possible

• Page 5 - It may be helpful to reflect on the consistency of the delivery of training i.e., provided by the same instructors, competencies of the instructors etc.

• Page 5, Line 25 – Spanish subtitles were used for the Australian videos. Consider clarifying the process in which MHFA suitability was adapted to ensure its relevance/ vaidity to a Latin American audience.

**Reviewer**	**2**
**Name**	**Gupta, Meena**
**Affiliation**	**Australian Catholic University, Library**
**Date**	**19-Nov-2025**
**COI**	**None**

Thank you for this topical submission

**Reviewer**	**3**
**Name**	**Naderimagham, Shohreh**
**Affiliation**	**Tehran University of Medical Sciences, Elderly Research Health Center**
**Date**	**13-Dec-2025**
**COI**	**None**

Mental Health First Aid training for Chile and Argentina: Protocol for a cluster randomised controlled trial

This is an important protocol addressing the cultural adaptation and evaluation of Mental Health First Aid (MHFA) training in two Latin American countries. It's relatively well designed, and the intervention is appropriate. However, several methodological details require clarification, and some sections are too descriptive for a protocol paper. Strengths & limitations are also not fully aligned with the BMJ Open expectations.

The manuscript would benefit from clearer justification of key design choices and more explicit methodological detail. It needs major revision before publication.

<u>MAJOR COMMENTS</u>

1. Cluster definition and structure need clearer explanation:

The manuscript claims that 12 clusters will be included, but the exact structure of the clusters, their average/expected size, and how they are paired and allocated are unclear, and clarification is necessary. It is not explicit whether the cluster size is fixed or whether

variables are only targeted. BMJ Open requires a table listing:

- number of clusters per arm
- assumptions about cluster size
- cluster types
- pairing method

2. Potential contamination between the intervention and control groups is not addressed:

Since clusters will be selected from universities, healthcare settings, and workplaces, maybe members of different arms will frequently interact. This raises a high risk of contamination, particularly for an educational intervention.

You should specify:

- expected contamination types
- minimization strategies
- plans for sensitivity analysis

3. The reasoning for selecting the three settings is not fully explained:

A more explicit justification is needed; otherwise, it remains unclear:

- Explain exactly why you chose these settings?
- Will you use convenience samples?
- Are the samples representative of broader community environments in Chile and Argentina?
- Whether organizational interests influence participant characteristics?

4. Sample size calculation doesn't have methodological transparency:

Although parameters such as ICC, effect size, and attrition assumption are reported, the actual calculation formulas, model assumptions, and software details are not presented. Sample size must explicitly state:

- number of clusters used in the calculation
- assumed equality of cluster size or variability
- reasoning behind the intra-cluster correlation
- source for effect size
- software used for analysis (e.g., Stata, R package)

5. The missing data strategy is incomplete:

You mentioned logistic regression to determine factors associated with missingness, but:

- There is no description of the imputation approach,
- There is no description of sensitivity analysis,
- There is no specification of the method for handling missing outcomes at T2/T3
- An anticipated 30% attrition is really a major limitation.

6. Blinding limitations are acknowledged, but bias-reduction procedures are not:

Since participants and staff cannot be blinded, the protocol should describe:

- How will performance bias be minimized?
- Which steps will reduce detection bias?
- How will you maintain statisticians' blinding throughout the study?

- How will self-report bias be monitored?

7. The cultural adaptation section is very long and descriptive:

This section focuses on results presentation rather than a protocol description! Since it contains:

- detailed pilot results,
- percentages,
- extensive narrative,
- statements from participants about usefulness, novelty, duration, and other features! BMJ Open recommends a brief description of intervention development, with complete details in supplementary files.

8. The study includes a high number of outcome measures:

The study has at least eight secondary outcomes. A justification for including so many outcomes, or a prioritization plan, is necessary. This may increase:

- respondent burden,
- risk of participant fatigue,
- potential reduction in data quality,
- increased risk of Type I error.

9. The trial timeline is missing:

SPIRIT guidelines require a table or figure showing:

- preparation phase,
- recruitment,
- intervention delivery,
- each follow-up period,
- analysis

timeline. This is not included.

10. Translation methodology requires more transparency:

The translation/back-translation process is described generally, but it lacks:

- What are the procedures for resolving discrepancies?
- Who made final decisions on disagreements?
- Were cognitive interviews conducted?
- Whether independent pilot testing of the vignettes was conducted?

MINOR COMMENTS

1. The Strengths and Limitations section needs more critical limitations:

Currently, the section:

- overstates strengths,
- lacks specific methodological limitations,
- does not include cluster-related challenges

BMJ Open recommends at least 1–2 real methodological limitations.

2. Several grammatical, spelling, and typographical problems:

Examples:

- "Stengths" must be "Strengths."
- "experimenting depression" must be "experiencing depression."
- Several sentences lack proper punctuation.
- Some compound words are missing hyphens (e.g., "real world" must be "real-world")

3. CONSORT/SPIRIT statements:
The manuscript should clearly mention:
"The SPIRIT checklist is provided as supplementary material."

4. The ethics section should be shorter:
There is no need to include details such as document tables, file names, or internal correspondence in the main protocol.

5. The caption for Figure 1 is too brief:
The caption should be expanded to summarize the study flow.

6. The introduction is very long:
The historical background of Latin American community mental health reform (1960s–1970s) is informative, but it is more detailed than necessary for a protocol.

Overall Recommendation: MAJOR REVISION

To improve the manuscript, the authors must address the reviewers' comments to make the article publishable.

VERSION 1 - AUTHOR RESPONSE

Response to reviewers

We thank the reviewers for their careful reading of our manuscript and their valuable insights, comments and suggestions that will give us the opportunity to improve it.

Answers to Reviewer 1:

This protocol is generally clear in its adherence to SPIRIT reporting guidelines and its potential impact within the mental health and academic space.

We thank the reviewer for this positive comment.

• Page 2 – Misspelling of strengths in 'strengths and limitations of this study'.

This has been corrected.

• Page 2- some reflection on the potential limitations on the study and how they would be mitigated could add to the richness of this protocol.

We have made changes to page 2 as follows:

- Cross-country comparisons should be interpreted with caution due to contextual and health system differences between countries.
- Individuals with limited literacy will not be included, as the study relies on self-completed questionnaires, which may limit generalisability.

And we have added mitigation strategies to potential problems during the study in pages 5 C 6.

• Introduction page 2, Line 34 – would be good to mention the types of programs that are/were available, what they offer, their measurable impacts (if any) and how they contribute to mental health care.

We have made the following changes to page 2 to reflect that there are not measurable impacts available:

"Adding to this, they have established programmes aiming to enhance suicide prevention[9] and to tackle the psychological consequences of potentially traumatic events.[10-12] However, these strategies, as in other countries, have not been properly evaluated and face multiple hurdles that coexist with high levels of stigma toward individuals with mental health problems, persistent treatment gaps, and the limited sensitivity of healthcare services to individuals' needs and preferences. This situation calls for additional progress and fresh initiatives."

However, as requested by Reviewer #3, a part of this paragraph was cut from the revised version to make the Introduction somewhat shorter.

• Page 3 Line 28 – embed the link to MHFA as a reference, if possible.

This has been added.

• Page 5 - It may be helpful to reflect on the consistency of the delivery of training i.e., provided by the same instructors, competencies of the instructors etc. We have added the following to page 5:

"Trainers from both countries, all with a background in clinical psychology, jointly received initial training and supervision from CK and NR during the materials revision

process and committed to delivering all courses in pairs in their respective countries throughout the study. In addition, a fidelity assessment tool was developed by the local teams, in consultation with NR and the MHFA International representative (CK), to ensure comparability across courses and between sites."

• Page 5, Line 25 – Spanish subtitles were used for the Australian videos. Consider clarifying the process in which MHFA suitability was adapted to ensure its relevance/ vaidity to a Latin American audience. We have added the following to page 5:

"Spanish subtitles were also created for seven of the Australian MHFA videos to ensure accessibility for participants who do not speak English."

Response to Reviewer #3

MAJOR COMMENTS

1. Cluster definition and structure need clearer explanation:

The manuscript claims that 12 clusters will be included, but the exact structure of the clusters, their average/expected size, and how they are paired and allocated are unclear, and clarification is necessary. It is not explicit whether the cluster size is fixed or whether variables are only targeted. BMJ Open requires a table listing:

- number of clusters per arm
- assumptions about cluster size
- cluster types
- pairing method

The following information has been added into the Trial schema and in the text on page 6.

"Each cluster is expected to include approximately 20 participants. No formal pairing criteria will be applied; however, the relative homogeneity of each setting and consideration of education level are intended to contribute to comparability between groups. In addition, potential contamination of groups will be considered (e.g., participants in the intervention group sharing what they learnt or training materials with participants in the control group). For example, students pursuing the same degree will be allocated to the same cluster, and participants from the same (or different) areas or facilities will likewise be grouped accordingly, in order to minimise the possibility of participants from different groups having regular contact in the course of their study or work. A summary of the number of clusters per arm, assumed cluster size, cluster types, and pairing approach is provided in Supplmentary Table 1."

A table listing this information has been included as Supplmentary material.

2. Potential contamination between the intervention and control groups is not addressed:

Since clusters will be selected from universities, healthcare settings, and workplaces, maybe members of different arms will frequently interact. This raises a high risk of contamination, particularly for an educational intervention.
You should specify:

- expected contamination types
- minimization strategies
- plans for sensitivity analysis

We have included the following information in two different sections (Recruitment of clusters on page 6, as mentioned above, and Data collection and measures on page 7).

"Participants in the intervention group will be advised not to share training materials (e.g., the training manual) with others for at least six months as they may be unaware whether those individuals are allocated to the control group. Participants in the wait-list arm will be explicitly asked about any prior exposure to the training curriculum before receiving the training; the proportion of participants reporting such exposure and its distribution across clusters will be considered in the interpretation of the findings."

3. The reasoning for selecting the three settings is not fully explained:

A more explicit justification is needed; otherwise, it remains unclear:

- Explain exactly why you chose these settings?
- Will you use convenience samples?
- Are the samples representative of broader community environments in Chile and Argentina?
- Whether organizational interests influence participant characteristics?

We thank the reviewer for raising these thoughtful questions. The following explanation of the rationale behind selecting these settings has been added to the Study design section on page 4.
"The settings were selected because they represent contexts in which community members are likely to encounter individuals experiencing mental health problems, and because MHFA is commonly implemented in similar settings internationally, supporting future scalability and implementation."

The use of convenience sampling is explicitly mentioned now in the Randomisation section on page 5.
"Clusters will be matched by setting (university, healthcare, or workplace) and then

randomly allocated to either the intervention or control condition. Local trial managers in Argentina and Chile will be responsible for recruiting participants into their respective clusters, using convenience sampling and taking into account degree programs and areas of employment to achieve a reasonable balance in years of education, so that participants in the intervention and the wait-list groups are as comparable as possible."

The strategy intended to minimise the influence of organisational interests has been added to page 6.
"Multiple meetings with representatives from each setting are planned in order to minimise the influence of organisational interests on the recruitment of participants."

4. Sample size calculation doesn't have methodological transparency:

Although parameters such as ICC, effect size, and attrition assumption are reported, the actual calculation formulas, model assumptions, and software details are not presented. Sample size must explicitly state:

- number of clusters used in the calculation
- assumed equality of cluster size or variability
- reasoning behind the intra-cluster correlation
- source for effect size
- software used for analysis (e.g., Stata, R package)

The following has been added to page 10:

"According to the Shiny CRT calculator, with 6 clusters per arm (12 in total) we will have 90% power, at a 2-sided 5% significance level, to detect a 0.5 standardised difference in mean intentions scores between intervention and control group at 6- month follow-up. In the absence of pilot data, we have assumed an ICC of 0.05, equal cluster sizes, a correlation of 0.5 between individual measurements over time and a 30% loss to follow-up. This effect size is based on a meta-analysis of MHFA training, which found pooled effect sizes for intentions were d=0.75 post training and d=0.55 up to six months after training."

Stata 19, the software to be used in the analysis, has been included in the Statistical analysis section.

5. The missing data strategy is incomplete:

You mentioned logistic regression to determine factors associated with missingness, but:

- There is no description of the imputation approach,
- There is no description of sensitivity analysis,
- There is no specification of the method for handling missing outcomes at T2/T3
- An anticipated 30% attrition is really a major limitation.

Mixed models do not require imputation of missing data; rather they use maximum likelihood estimation to accommodate missing observations under the assumption that

data are missing at random. We have edited the description on page 10 and 11 to be more explicit about this:

"This method takes into consideration the data's hierarchical structure (i.e. the correlation of measurement occasions within participants and within clusters) and uses maximum likelihood methods to generate unbiased estimates even when some participants have missing data, provided that the data are missing at random."

While 30% attrition may be high, it is a reasonable expectation given our experience conducting Delphi studies in preparation for this trial in Chile and Argentina, and we have been conservative in our assumptions so that we are not underpowered.

6. Blinding limitations are acknowledged, but bias-reduction procedures are not:

Since participants and staff cannot be blinded, the protocol should describe:

- How will performance bias be minimized?
- Which steps will reduce detection bias?
- How will you maintain statisticians' blinding throughout the study?
- How will self-report bias be monitored?

We have added the following two paragraphs in the Data collection and measures section on page 7.

"Performance bias will be minimised by ensuring that baseline (T1) questionnaires are completed before participants are informed of their allocation to the intervention or the control group. In addition, invitations and reminders will be identical across groups at all time points. At T2 and T3, participants will be reminded to carefully complete the questionnaires. Research staff involved in participant contact will use standardised and neutral messaging with no differentiation by study arm. To reduce the influence of inattentive responding, responses times will be monitored. Questionnaires completed in less than 5 minutes (approximately a quarter of the anticipated average completion time of 20 minutes) will be flagged.
The proportion of such responses and their distribution across study arms and time points will be reported, and their potential influence on the results will be explored.

Outcomes are assessed using self-report measures, and self-report bias (e.g., social desirability) cannot be ruled out. However, the instruments have been previously piloted and used in similar populations, demonstrating acceptable reliability.[38]
Repeated measurements over time will also allow the examination of consistency in response patterns, which will be reported."

Regarding the statisticians' blinding we have added the following to the Data monitoring and management and the Statistical analyses sections on page 11.

"However, NR and AM will remain blind to arm allocation until completion of the statistical analysis."

"Statistical analyses will be conducted by a researcher not involved in participant recruitment or intervention delivery and blinded to allocation, following intention- to-treat principles; all participants who consent to participation will be included in the analysis, regardless of intervention completion or follow-up responses."

7. The cultural adaptation section is very long and descriptive:

This section focuses on results presentation rather than a protocol description! Since it contains:

- detailed pilot results,
- percentages,
- extensive narrative,
- statements from participants about usefulness, novelty, duration, and other features! BMJ Open recommends a brief description of intervention development, with complete details in supplementary files.

We have significantly reduced this section this information is now presented in a single and shorter paragraph on page 5.

8. The study includes a high number of outcome measures:

The study has at least eight secondary outcomes. A justification for including so many outcomes, or a prioritization plan, is necessary. This may increase:

- respondent burden,
- risk of participant fatigue,
- potential reduction in data quality,
- increased risk of Type I error.

Evaluations of MHFA training often have a large number of outcome measures because the concept of mental health literacy has multiple components, which are measured with different questionnaires. While there may be several measures, we have chosen them with care to reduce participant burden, such that the surveys take 15 to 20 minutes to complete. We have used these questionnaires successfully in a recent CRCT of MHFA training in China and including them here also allows for cross-country comparisons. We have prioritised the primary outcome to be intended behaviour, as is best practice.

The following paragraph was added to page 10.

"The selection of intentions to provide mental health first aid as the primary outcome is consistent with prior evaluations of MHFA training.[32 52] In line with MHFA being conceptualised as an intervention targeting mental health literacy, which comprises multiple distinct components,[26 53] several secondary outcomes are included to capture these dimensions. These secondary outcomes will be treated as exploratory, and findings will be interpreted cautiously in light of the increased risk of Type I error associated with multiple comparisons."

9. The trial timeline is missing:

SPIRIT guidelines require a table or figure showing:

- preparation phase,
- recruitment,
- intervention delivery,
- each follow-up period,
- analysis timeline. This is not included.

We have added the trial timeline. Please see figure 2.

10. Translation methodology requires more transparency:

The translation/back-translation process is described generally, but it lacks:

- What are the procedures for resolving discrepancies?
- Who made final decisions on disagreements?
- Were cognitive interviews conducted?
- Whether independent pilot testing of the vignettes was conducted?

We have modified the section on page 8, which now states:

To ensure accuracy of translation, the vignette was translated into Spanish independently by two bilingual translators (native Spanish speakers); the translations were compared item by item and, after reaching consensus, were back- translated into English by a bilingual native English speaker. The back-translation was compared with the original version and no substantive differences were identified. Minor discrepancies were discussed as a group and the final Spanish version was agreed by consensus among all bilingual translators involved in this process.

Given the high level of disability associated with psychosis and the critical importance of its early detection, a vignette describing a person ("A") with psychotic symptoms consistent with DSM-5 criteria for schizophrenia, which underwent an identical translation process, will also be used, with responses coded as 'correct' if

they include mention of "psychosis" or "schizophrenia". Cognitive interviewing was not conducted as a separate step; however, the vignettes were reviewed by the research team and administered during pilot testing of the course, during which no major difficulties in comprehension were identified. Both vignettes will use a gender-neutral format.

MINOR COMMENTS

1. The Strengths and Limitations section needs more critical limitations:

Currently, the section:

- overstates strengths,
- lacks specific methodological limitations,
- does not include cluster-related challenges

BMJ Open recommends at least 1–2 real methodological limitations.

The following has been added to page 2:

2. Several grammatical, spelling, and typographical problems:

Examples:

- "Stengths" must be "Strengths."
- "experimenting depression" must be "experiencing depression."
- Several sentences lack proper punctuation.
- Some compound words are missing hyphens (e.g., "real world" must be "real-world")

These have been corrected.

3. CONSORT/SPIRIT statements:

The manuscript should clearly mention:
"The SPIRIT checklist is provided as supplementary material."

The following has been added to page 3:

4. The ethics section should be shorter:

There is no need to include details such as document tables, file names, or internal correspondence in the main protocol.

These have been corrected.

5. The caption for Figure 1 is too brief:

The caption should be expanded to summarize the study flow.

The figure caption has been expanded and now reads: Figure 1: Trial schema showing cluster randomisation and assessment timepoints for both arms

6. The introduction is very long:

The historical background of Latin American community mental health reform (1960s–1970s) is informative, but it is more detailed than necessary for a protocol.

We have summarised this section according to the suggestion.

Overall Recommendation: MAJOR REVISION

To improve the manuscript, the authors must address the reviewers' comments to make the article publishable.
